# Influence of Tumour Necrosis Factor Alpha on the Outcome of Ischaemic Postconditioning in the Presence of Obesity and Diabetes

**DOI:** 10.1155/2012/502654

**Published:** 2012-10-17

**Authors:** Lydia Lacerda, Lionel H. Opie, Sandrine Lecour

**Affiliations:** Cardioprotection Group, Hatter Institute for Cardiovascular Research in Africa, Department of Medicine, Faculty of Health Sciences, University of Cape Town, Anzio Road, Observatory, Cape Town 7925, South Africa

## Abstract

Obesity and diabetes contribute to cardiovascular disease and alter cytokine profile. The cytokine, tumour necrosis factor alpha (TNF**α**), activates a protective signalling cascade during ischaemic postconditioning (IPostC). However, most successful clinical studies with IPostC have not included obese and/or diabetic patients. We aimed to investigate the influence of TNF**α** on the outcome of IPostC in obese or diabetic mice. TNF knockout or wildtype mice were fed for 11 weeks with a high carbohydrate diet (HCD) to induce modest obesity. Diabetes was induced in a separate group by administration of a single intraperitoneal injection of streptozotocin. Hearts were then isolated and subjected to ischaemia (35 min of global ischaemia) followed by 45 min of reperfusion. HCD increased body weight, plasma insulin and leptin levels while the glucose level was unchanged. In streptozotocin-treated mice, blood glucose, plasma leptin and insulin were altered. Control, obese or diabetic mice were protected with IPostC in wiltype animals. In TNF knockout mice, IPostC failed to protect control and diabetic hearts while a slight protection was observed in obese hearts. Our data confirm a bidirectional role for TNF**α** associated with the severity of concomitant comorbidities and suggest that diabetic and/or modestly obese patients may still benefit from IPostC.

## 1. Introduction

Both obesity and diabetes are major risk factors for cardiovascular disease. Forty years ago, fewer than 25% of adults in the USA were classified as overweight or obese compared with 75% in 2002 [1–3]. Because the onset of type1 diabetes occurs at a young age, the cardiovascular risk is increased tenfold when compared with nondiabetic peers [4]. In addition, obesity and diabetes are associated with an increased mortality and an attenuation of tolerance to ischaemic events [5, 6]. 

Experimental data suggest that the protective effect of ischaemic postconditioning (IPostC) (defined as a series of brief episodes of alternating reperfusion and ischaemia at the onset of reperfusion) is diminished in animals with comorbidities such as obesity and diabetes; see review [7]. Bouhidel demonstrated that the protective effect of IPostC against reperfusion injury in ob/ob mice was impaired [8]. Furthermore, obesity and diabetes compromise the inflammatory system with altered expression of tumour necrosis factor alpha (TNF*α*) occurring in adipose and muscle tissue of obese humans [9]. However, whether this alteration is beneficial or deleterious to the heart still remains unclear. The expression of adipokines, such as leptin, is also modified in obesity and diabetes and there is a strong correlation between serum leptin and TNF*α* levels [10]. Additionally, leptin has been reported to protect against lethal reperfusion injury in the isolated mouse heart via direct action on the heart [11, 12].

A dual role for TNF*α* in the heart has been postulated whereby beneficial effects are seen at low concentrations and deleterious effects become evident at higher concentrations in a time-dependent manner [13–15]. TNF*α* can activate both TNF receptor 1 and TNF receptor 2 which seem to exert opposite effects in the heart [16]. In a mouse model, we have recently shown that a low concentration of TNF*α* activates a protective signalling cascade during IPostC via the activation of the TNF receptor 2 [17]. However, the outcome of IPostC in obese and diabetic patients remains uncertain as the recent application of this therapy in clinical studies has excluded patients with such comorbidities.

In the present study, we aimed to investigate the influence of TNF*α* on the outcome of IPostC in obese or diabetic mice.

## 2. Methods

All animal studies performed were approved by the Animal Research Ethics Committee of the University of Cape Town and followed the recommendations laid down in the Guide for the Care and Use of Laboratory Animals published by the US National Institute of Health (NIH Publication no. 85-23, revised 1996).

### 2.1. High-Carbohydrate Diet (HCD) Mouse Model

Wild-type (TNF-WT) and TNF*α* knockout (TNF^−^/^−^) mice were separated into 2 groups each, with 6 mice per group. One group of TNF-WT and one group of TNF^−^/^−^ mice each received a normal diet (ND) of mouse chow and the second groups of TNF-WT and TNF^−^/^−^ each received a diet containing elevated carbohydrates and fats mimicking a Western-type diet (HCD), for 11 weeks as illustrated in Table 1 [5].

### 2.2. Streptozotocin Diabetic Mouse Model (STZ)

Experimental diabetes type 1 was induced in a total of 10 mice per group by a single intraperitoneal (i.p.) injection of 180 mg streptozotocin (STZ)/kg body weight, dissolved in 0.1 mol citrate buffer [18]. Nondiabetic control animals were treated with solvent (citrate buffer) alone. Standard mouse chow and tap water were provided ad libitum for all groups. At the end of each time period, only STZ-treated mice with a blood glucose level greater than 16 mmol/L were considered as diabetic (normal blood glucose levels in mice range from 3.4 to 9.7 mmol/L). Of the 10 STZ-treated TNF-WT mice, 7 met the criteria, 2 did not achieve sufficiently high glucose levels, and 1 died shortly after receiving the STZ. No death occurred in the 10 Nondiabetic control animals in each group.

### 2.3. Blood Glucose and Glucose Tolerance Test

Blood glucose and the glucose tolerance test (GTT) were done at 14 weeks. Mice were fed normal chow or HCD as described in Table 1. Prior to performing the blood glucose tolerance test, the mice were fasted for 7 hours, but had free access to drinking water. A 20% solution (w/v) of glucose was made up in sterile distilled water. Each mouse was then sedated with a mixture of ketamine (75 mg/kg) and medetomidine (0.5 mg/kg) given i.p. Body weight was recorded for each mouse; a tail cut was done and blood glucose determined in mmol/L by means of a blood glucose monitor (Accu-Chek Active; Roche Diagnostics, Mannheim, Germany) as per the manufacturer's instructions. A bolus of glucose was given i.p. (15 mg/kg). Thereafter, a blood glucose measurement was performed every 30 min after injection, until a decrease in the glucose level was observed (120 to 150 min). 

### 2.4. Perfusion of Mouse Hearts

The HCD-fed mice (14 weeks of age), the STZ-treated mice (5 and 10 days after STZ treatment), and their respective controls were anaesthetized (sodium pentobarbitone, 60 mg/kg i.p.) and heparinized (25 IU i.p.). Hearts were isolated and perfused retrogradely as previously described [19]. At the same time, blood was taken from the thoracic cavity of each mouse and placed in a chilled heparinized tube, centrifuged at 5000 rpm for 5 min at 4 degrees. The plasma was removed and frozen for further analysis.

### 2.5. Ischaemic Postconditioning

HCD-fed mice and STZ-treated mice were subjected to the ischaemic postconditioning (IPostC) protocol which consisted of six alternating cycles of 10-second reperfusion, 10-second ischaemia, commencing at the onset of reperfusion as described previously [17].

### 2.6. Ratio of Heart Weight to Body Weight

At the end of the perfusion protocol, each heart was carefully dried and weighed after staining with *triphenyl tetrazolium chloride* (TTC). The heart weight to body weight ratio for each mouse was then calculated.

### 2.7. Insulin Levels

Quantitative determination of baseline insulin levels was performed using the Ultrasensitive Mouse Insulin Elisa Kit (Crystal Chem Inc.; USA) as per the manufacturer's instructions. 

### 2.8. Leptin Levels

Quantitative determination of baseline leptin levels was performed using the Ultrasensitive Mouse Leptin Elisa Kit (Crystal Chem Inc.; USA) as per the manufacturer's instructions.

### 2.9. Chemicals and Pharmacological Agents

Unless otherwise stated, all chemicals were obtained from Sigma-Aldrich Chemicals, Germany.

### 2.10. Statistical Analysis

Data are presented as mean ± SEM. Comparisons between multiple groups were performed by 1-way ANOVA followed by Tukey post hoc test or Bonferroni multiple comparison test (GraphPad Instat). Two-way ANOVA followed by Bonferroni multiple comparison test (GraphPad Prism) was performed where species or diet differed. *P* < 0.05 was considered to be statistically significant.

## 3. Results

### 3.1. Effect of High Carbohydrate Diet on Physiological Parameters in TNF-WT Mice

**Figure 1:**
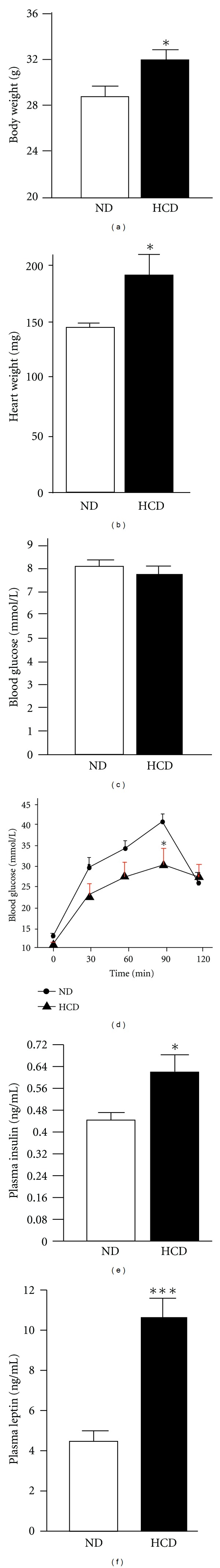
*Effects of high carbohydrate diet (HCD) on physiological parameters in TNF-WT mice*. HCD increased body weight (a), heart weight (b), plasma insulin (e) and plasma leptin levels (f), decreased blood glucose tolerance test (d) but no significant difference was observed on blood glucose levels (c), *P < 0.05; ***P < 0.001 versus normal diet (ND); *n* ≥ 6 for all groups.

HCD increased the body and heart weights in the TNF-WT mice from 29.0 ± 1.0 grams to 32.0 ± 0.7 grams (*P* < 0.05 versus ND; Figures 1(a) and 1(b)). Although HCD did not affect baseline blood glucose levels (*P* = ns, Figure 1(c)) it improved glucose tolerance in the WT animals (*P* < 0.05 versus ND, Figure 1(d)) and increased plasma insulin and leptin levels to 0.64 ± 0.06 ng/mL from 0.46 ± 0.03 ng/mL in ND (*P* < 0.05) and to 10.6 ± 0.9 ng/mL from 4.5 ± 0.4 ng/mL in ND (*P* < 0.001, resp., Figures 1(e) and 1(f)).

### 3.2. Effect of High Carbohydrate Diet on IPostC-Induced Cardioprotection in TNF-WT Hearts

**Figure 2:**
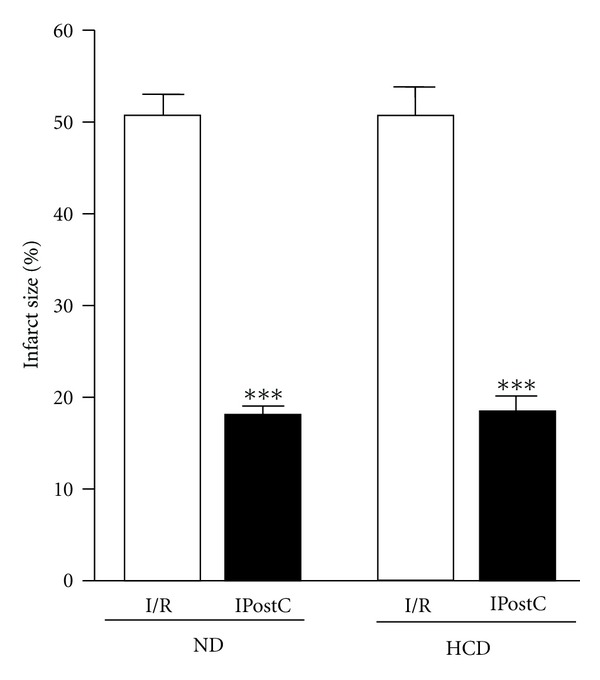
*Effect of obesity on IPostC-induced cardioprotection in isolated TNF-WT hearts*. IPostC in TNF-WT with normal diet (ND) reduced infarct size from 50 ± 2% in the ischaemic control group (I/R) to 18 ± 1% for IPostC. The infarct size in TNF-WT with high carbohydrate diet (HCD) was decreased to 19 ± 2% versus 50 ± 2% for the I/R group, ***P < 0.001 versus I/R; *n* ≥ 6.

Mice on the ND presented a similar infarct size to those fed an HCD mice when subjected to I/R (*P* = ns). IPostC reduced the infarct size to a similar extent in ND and HCD mice versus their respective ischaemia-reperfusion control groups (*P* < 0.001) (Figure 2).

### 3.3. Effect of High Carbohydrate Diet on Physiological Parameters in TNF^−^/^−^ Mice

**Figure 3:**
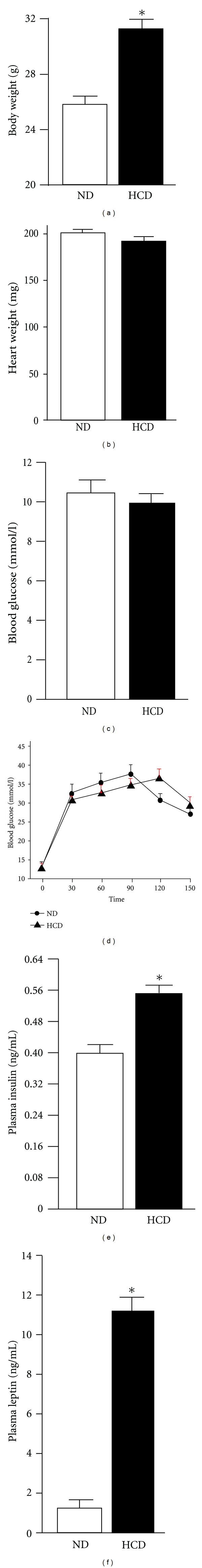
*Effects of obesity on physiological parameters in TNF* ^−/−^ *mice*. High carbohydrate diet (HCD) increased body weight (a), plasma insulin (e) and plasma leptin levels, whereas blood glucose levels (c) and heart weight (b) remained unchanged. *P < 0.05 versus normal diet (ND); *n* = 6 for all groups.

TNF^−^/^−^ mice were used to investigate the role of TNF*α* in obesity. Similar to TNF-WT mice, body weight was increased with HCD from 26.0 ± 0.4 grams to 31.5 ± 0.5 grams (*P* < 0.05 versus ND, Figure 3(a)). However, there was no change in heart weight in TNF^−^/^−^ mice on the HCD compared to their ND counterparts (*P* = ns, Figure 3(b)).

Baseline blood glucose and tolerance of HCD-fed TNF-deficient mice to glucose remained unchanged by HCD, (*P* = ns; Figures 3(c) and 3(d)). Plasma insulin levels were increased significantly by HCD from 0.41 ± 0.02 ng/mL to 0.57 ± 0.01 ng/mL (*P* < 0.05 versus ND, Figure 3(e)). Similarly, the plasma leptin levels were significantly increased by the HCD from 1.3 ± 0.08 ng/mL to 11.2 ± 0.8 ng/mL (*P* < 0.05 versus ND, Figure 3(f)).

### 3.4. Effect of High Carbohydrate Diet on IPostC-Induced Cardioprotection of TNF^−^/^−^ Hearts

**Figure 4:**
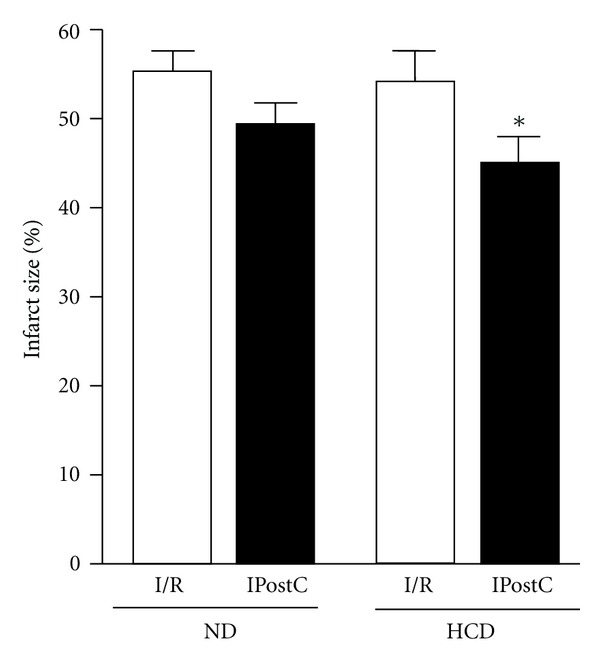
*Effect of obesity on IPostC-induced cardioprotection in isolated TNF* ^−/−^ *mice*. IPostC reduced infarct size in the HCD fed mice from 56 ± 2% to 45 ± 4% whereas mice fed the normal diet could not be protected, *P < 0.05 versus I/R; *n* ≥ 6.

To determine whether absence of TNF*α* in obesity can affect the outcome of IPostC, the isolated hearts of TNF^−^/^−^ mice fed with either a normal diet or an HCD were subjected to the IPostC protocol. HCD mice subjected to I/R presented a similar infarct size compared to mice fed with ND (*P* = ns). Surprisingly, the hearts from TNF^−^/^−^ mice fed with HCD demonstrated a slight reduction in infarct size versus the I/R control (*P* < 0.05; Figure 4).

### 3.5. Effects of Diabetes on Physiological Parameters in TNF-WT Mice

**Figure 5:**
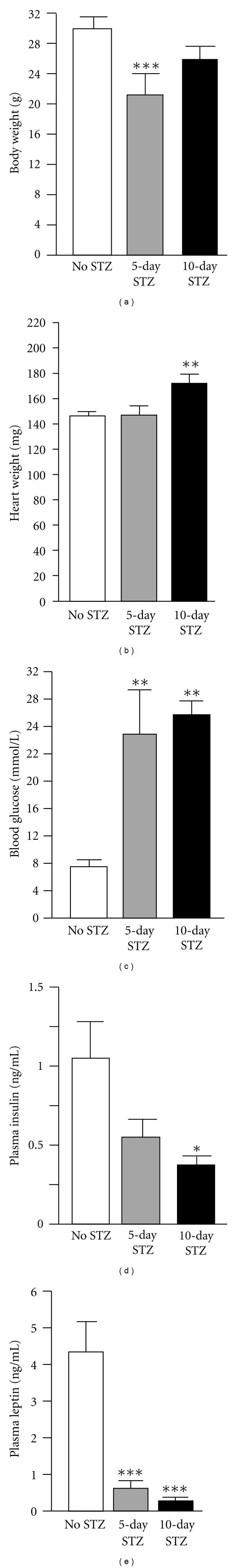
*Effects of diabetes on physiological parameters in TNF-WT mice*. Body weight was decreased 5 days post STZ treatment and returned to normal at day 10 post STZ treatment (a). Heart weight was reduced 10 days post STZ treatment (b). Insulin and leptin levels were both decreased with post STZ treatment (d and e) while STZ increased baseline blood glucose at days 5 and 10, *P < 0.05 and ***P < 0.001 versus no STZ.

To create a type 1 diabetic model, TNF-WT mice were given a single intraperitoneal injection of streptozotocin (180 mg/kg body weight). Physiological parameters and experiments were performed either 5 days or 10 days after STZ administration. TNF-WT mice had a significant decrease in body weight 5 days after treatment, from 29.7 ± 1.3 grams to 21.0 ± 2.6 grams (*P* < 0.001 versus no STZ). However, the weight was restored by day 10 (*P* = ns versus no STZ; Figure 5(a)). There was no change in the heart weight after 5 days of STZ (*P* = ns; Figure 5(b)), whereas, after 10 days after STZ treatment, the heart weight was significantly increased (*P* < 0.01 versus untreated controls). Streptozotocin injection increased baseline blood glucose at days 5 and 10 (*P* < 0.01 versus no STZ; Figure 5(c)), but decreased plasma insulin from 1.02 ± 0.2 ng/mL to 0.53 ± 0.14 ng/mL (5 days after STZ) and to 0.36 ± 0.02 ng/mL (10 days after STZ) (*P* < 0.05 versus no STZ-treatment; Figure 5(d)). Similar results for glucose and insulin after streptozotocin treatment have been reported [20]. Leptin levels were also reduced by STZ at day 5 and 10 after injection from 4.5 ± 0.7 ng/mL to 0.6 ± 0.2 ng/mL and 0.23 ± 0.04 ng/mL, respectively (*P* < 0.001 versus no STZ treatment; Figure 5(e)).

### 3.6. Effect of Diabetes on IPostC-Induced Cardioprotection in TNF-WT Hearts

**Figure 6:**
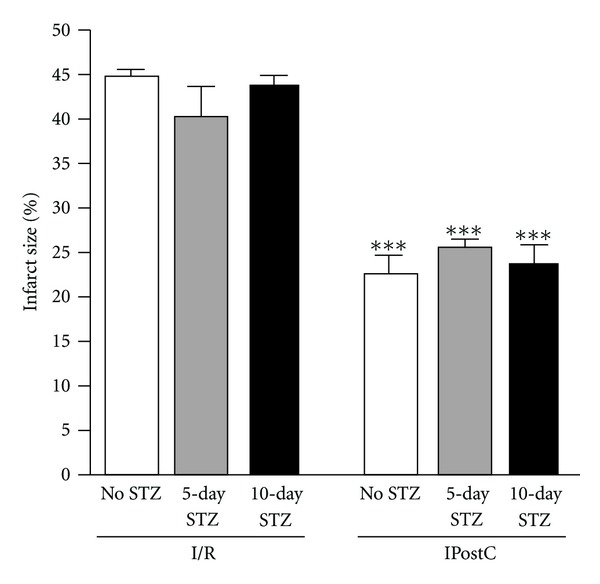
*Effect of diabetes on IPostC induced cardioprotecion in isolated TNF-WT mice*. Infarct size was significantly decreased by IPostC in control and streptozotocin treated animals, ***P < 0.001 versus respective I/R controls.

STZ-treated TNF-WT mice subjected to I/R showed a similar infarct size to the untreated I/R control, at both 5 days and 10 days after treatment (*P* < 0.001 versus I/R). STZ treatment did not affect the cardioprotective effect of IPostC after 5 or 10 days versus the untreated animals, (*P* < 0.001; Figure 6).

### 3.7. Effect of Diabetes on Physiological Parameters in TNF^−^/^−^ Mice

**Figure 7:**
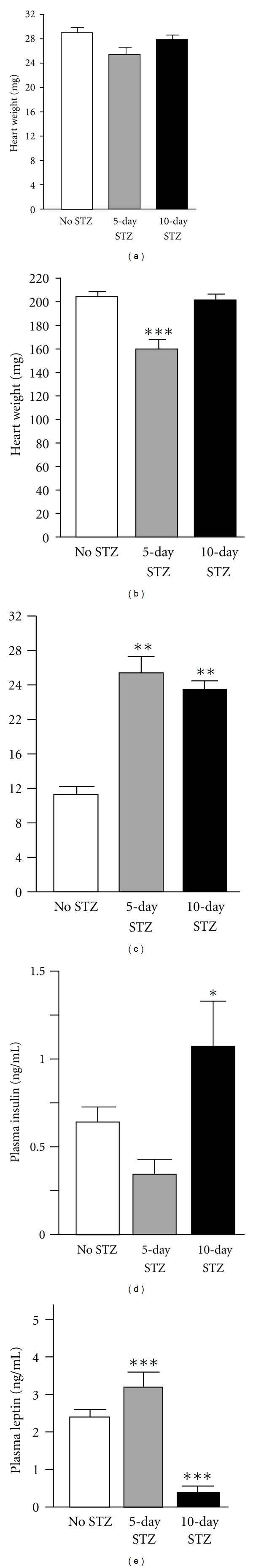
*Effects of diabetes on physiological parameters in TNF* ^−/−^ *mice*. Body weight remained unchanged in Streptozotocin (STZ)-treated mice (a) but decreased the heart weight 5 days post treatment (b). mice (a) but decreased the heart weight 5 days post treatment (b). Blood glucose (c), plasma insulin (d) and plasma leptin (e) were increased with STZ, *P < 0.05, **P < 0.01, ***P < 0.001 versus no STZ; *n* = 6.

To investigate whether TNF*α* plays a role in type 1 diabetes and cardiovascular disease, TNF*α*-deficient mice were injected intraperitoneally with a single dose of streptozotocin (180 mg/kg body weight). Similar to TNF-WT mice, STZ administration had no effect on body weight, 5 or 10 days after STZ treatment (*P* = ns, Figure 7(a)). However, the heart weight was significantly decreased by the STZ treatment after 5 days (*P* < 0.001 versus untreated, Figure 7(b)) but 10 days after treatment the heart weight was similar to untreated controls (*P* = ns versus untreated, Figure 7(b)). As expected, STZ increased baseline blood glucose at both time points (*P* < 0.001 versus untreated, Figure 7(c)). Although no significant difference was seen in plasma insulin levels at 5 days after STZ treatment, there was a significant increase 10 days after treatment, from 0.32 ± 0.1 ng/mL to 1.06 ± 0.3 ng/mL (*P* < 0.01 versus untreated control, Figure 7(d)). Similarly to the TNF-WT mice, the diabetic TNF^−^/^−^ animals demonstrated an elevated level of plasma leptin at 5 days after STZ administration, from 2.3 ± 0.1 ng/mL to 3.0 ± 0.4 (*P* < 0.001 versus untreated control), but the leptin level was drastically reduced in the 10-day post-treatment group to 0.3 ± 0.08 ng/mL (*P* < 0.05 versus untreated control group, Figure 7(e)).

### 3.8. Effect of Diabetes on IPostC-Induced Cardioprotection in TNF^−^/^−^ Hearts

**Figure 8:**
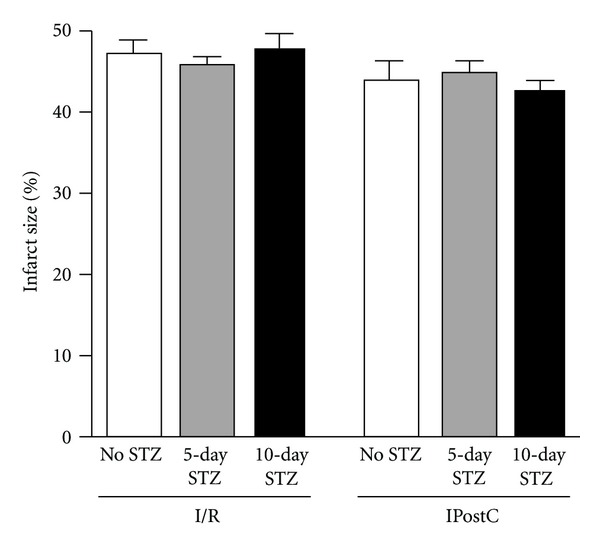
*Effect of diabetes on IPostC induced cardioprotecion in isolated TNF* ^−/−^ *mice*. Ischaemic postconditioning failed to protect hearts from TNF^−/−^ mice both at 5 and 10 days after STZ-treatment, *P* = ns; *n* = 6.

IPostC failed to confer protection in the STZ-treated TNF*α* knockout animals (*P* = ns versus I/R; Figure 8).

## 4. Discussion

Our data revealed that 11 weeks of a high-carbohydrate diet, or the administration of a single intraperitoneal injection of streptozotocin, resulted in a modest model of obesity or diabetes, as demonstrated by changes in body weight, blood glucose levels, plasma insulin, and plasma leptin levels. IPostC-induced cardioprotection was evident in the modestly obese TNF-WT mice and also in the diabetic TNF-WT mice, suggesting that the presence of obesity/diabetes did not alter the cardioprotective signalling cascade activated by IPostC. However, in the absence of TNF*α*, the IPostC stimulus did not protect the healthy and diabetic mice against I/R injury. Surprisingly, there was slight restoration of the cardioprotective effect in the modestly obese TNF^−^/^−^ animals, reinforcing the concept that TNF*α* has both deleterious and beneficial effects in the heart.

### 4.1. Obesity/Diabetes and Susceptibility to Ischaemia-Reperfusion

Many of the signalling cascades involved in cardioprotection may be affected by various factors such as preexisting disease, age, and cotreatments [21, 22]. To date, cardioprotective investigations have been performed mainly in young and healthy animals, which is far different from the clinical setting [23, 24]. The high-carbohydrate diet used in our study is of a similar composition than the conventional Western-type diet of humans and was chosen to represent a modestly obese phenotype [5], unlike the more severe obese models of either ob/ob mice or the db/db mice which are either leptin deficient or have no leptin receptors. In our model, high-carbohydrate diet did not affect the damage following ischemia-reperfusion. Obesity is associated with hyperinsulinaemia which markedly modulates the extent to which myocardial injury occurs during ischaemia-reperfusion [25]. Therefore, it is plausible to suggest that, in obesity, the impact of high levels of circulating insulin during ischaemia and reperfusion could overshadow myocardial susceptibility to ischaemia-reperfusion injury. 

### 4.2. Obesity/Diabetes Susceptibility to IPC and IPostC

Our data demonstrate that obesity, induced by a high-carbohydrate diet or diabetes, induced by injection of streptozotocin, did not affect the cardioprotective effect of IPostC in the wildtype animals. Failure of IPostC to limit infarct size was reported from a study conducted in ob/ob mice [8]. However, a limitation of this study was the lack of leptin in this mouse strain. A very recent study conducted in a murine model of streptozotocin-induced diabetes (using a similar dose to our study) reported a loss of efficacy in IPostC-induced cardioprotection [26]. Possible explanations for the contradiction between this study and our present findings are as follows: (1) the insulin levels in the mice of the published study were significantly lower (0.18 ± 0.08 ng/mL) than the insulin levels found in our diabetic mice (0.36 ± 0.02 ng/mL); (2) the difference in mouse species, (3) the difference in age of the animals, and (4) the number of I/R cycles performed to postcondition the heart. The ischemic postconditioning algorithm chosen may influence the postconditioning effect [27]. In our study, we have used 6 cycles while published studies that failed to postcondition the diabetic heart have used 3 cycles [26]. It is possible that the threshold of protection has been raised with 6 cycles. We have recently reported that age, strain, and the postconditioning algorithm are critical factors to consider for successful cardioprotection with postconditioning and a minute difference in age, for example, can lead to an opposite outcome [28].

### 4.3. TNF*α* and Myocardial Function

Although TNF*α* is known to have a detrimental effect in ischaemia-reperfusion [29], we have previously demonstrated that TNF*α* is required for the protection with ischaemic pre- and postconditioning [17, 30]. In fact, TNF*α* is cardioprotective in a dose- and time- dependent manner [31]. Depending on which TNF receptor is activated, TNF*α* can be either harmful or protective with the activation of the TNF receptor 1 being harmful and the activation of the receptor 2 being protective [17, 32]. The cardioprotective effect of TNF*α* initiates a prosurvival signalling cascade termed as the survivor activating factor enhancement (SAFE) pathway that involves the activation of the transcription factor STAT-3 and possibly the closure of the mitochondrial permeability transition pore [33, 34].

### 4.4. Role of TNF*α* in Obese and Diabetic Animals

The role of TNF*α* in diet-induced obesity may depend on the TNF receptors activated with TNF receptor 1 being deleterious and TNF receptor 2 being cardioprotective [35]. Our data showed that the body weight was increased by 21% in TNF^−^/^−^ mice fed with HCD versus only 11% in the TNF-WT mice subjected to the same regime, therefore suggesting a protective effect of TNF*α* in diet-induced obesity. It would be of interest to repeat our experiments in our TNF^−^/^−^, TNFR1^−^/^−^, and TNFR2^−^/^−^ animals to further delineate the role of TNF*α* receptors in our model. 

The presence of TNF*α* in obesity has been reported to contribute towards the development of cardiac hypertrophy in cardiomyocytes [35]. In support of this hypothesis, our data demonstrate an increase in the heart weight in the TNF-WT mice fed with an HCD whereas, in the absence of TNF*α*, the HCD had no effect on the heart weight.

In our obese and diabetic models, the absence of TNF*α* did not affect the damage in hearts subjected to ischemia-reperfusion. However, it is important to note that our ischemia-reperfusion insult was performed *in vitro* and it may not translate to an *in vivo* setting.

TNF*α* production is markedly increased in muscle and adipose tissue in obese humans and rodent models of obesity-diabetes, compared with tissues of lean individuals [36]. The risk of cardiac microvascular disease is also increased in the diabetic individual and the release of circulating microparticles may favour the release of TNF*α* from endothelial cells [37]. Several studies have demonstrated that TNF*α* plays a role in mediating insulin resistance as a result of obesity [38–40]. Three factors which contribute to the control of body weight have been linked to TNF*α*: (1) the intake of food, (2) expenditure of energy, and (3) storage of energy. 

Administration of TNF*α* in a rat model resulted in reduced food intake [41] and also inhibited gastric emptying, leading to a feeling of satiation, most likely due to activation of leptin [42, 43]. Neutralization of TNF*α* by intravenous administration of a soluble TNF receptor-immunoglobulin G chimeric protein provided a significant improvement in insulin sensitivity in fatty rats [38–40], but treatment of non-insulin-dependent diabetes mellitus patients with a specific TNF*α* antibody had no effect on insulin sensitivity [44].

Although TNF*α* has been proposed as a link between obesity and insulin resistance [45], the baseline blood glucose was unchanged by HCD in the TNF-WT and TNF^−^/^−^ mice in our study. However, plasma insulin levels were increased, therefore suggesting the development of insulin resistance, even in the absence of TNF*α*.

Surprisingly, the high carbohydrate diet slightly restored the protective effect of IPostC in the TNF-deficient mice, therefore suggesting that absence of TNF*α* in obesity may be of benefit to the heart. In our modestly obese TNF^−^/^−^ mice, the plasma leptin level was significantly elevated compared to the animals kept on the normal diet where IPostC-induced protection was abrogated, therefore suggesting that this adipokine may be implicated in a compensatory mechanism. Leptin has been demonstrated to exhibit direct cardioprotective effects by targeting the mitochondrial permeability transition pore [11]. It is possible that the increased level of leptin in obesity might in fact protect the individuals with a higher body mass index after a myocardial infarction [22, 46, 47].

The increased plasma leptin levels observed in our TNF^−^/^−^ mice correlate with an increase in body weight and the same correlation was found in mice fed with a high-fat diet [48]. In contrast to our obese TNF^−^/^−^ mice, our STZ-induced diabetic TNF-WT model had significantly decreased leptin levels and the protective effect of IPostC was not significant. It has recently been reported that the tissue-preserving actions of leptin are influenced by obesity [48]. Dixon's group showed that leptin decreased the infarct size in Wistar and Zucker lean rats, which have functional leptin receptors, but the cardioprotection was lost in the Zucker obese rats in which the leptin receptors are nonfunctional [49]. These data provided evidence suggesting that the tissue-preserving actions of leptin are influenced by the severe obesity seen in Zucker obese rats. Thus, the degree of obesity as well as the presence or absence of TNF*α* may be of importance in determining the protective effects of leptin.

## 5. Conclusion

In conclusion, our data demonstrate that the cardioprotective effect of IPostC was unaltered in a high-carbohydrate diet mouse model of obesity and streptozotocin-induced diabetes. Whilst TNF*α* is necessary for the maintenance of glucose homeostasis, for the control of appetite to prevent obesity and for IPostC-induced cardioprotection, it can also lead to cardiac hypertrophy. The absence of TNF in mice did not affect the outcome of obese and diabetic mice subjected to an ischemia-reperfusion insult. IpostC failed to protect in healthy or obese TNF^−^/^−^ mice. However, a slight protection with IPostC was observed in our TNF^−^/^−^ model in the presence of obesity, illustrating the bidirectional effect of TNF*α* in the heart and the fact that the role of TNF*α* in obesity- and diabetes-related ischaemic heart disease remains a complex system. Nevertheless, our data suggest that obese and type 1 diabetic individuals may still benefit from IPostC, relative to the severity of the disease.

## Figures and Tables

**Table 1:** Energy provided by high carbohydrate diet (HCD) versus normal chow (ND).

	Normal diet (ND)	High-carbohydrate diet (HCD)
Carbohydrates (%)	60	69
Proteins (%)	30	17
Fats (%)	10	14
